# Transfer learning for predicting of gross domestic product growth based on remittance inflows using RNN-LSTM hybrid model: a case study of The Gambia

**DOI:** 10.3389/frai.2025.1510341

**Published:** 2025-02-24

**Authors:** Haruna Jallow, Ronald Waweru Mwangi, Alieu Gibba, Herbert Imboga

**Affiliations:** ^1^Department of Mathematics (Data Science Option), Pan African University Institute for Basic Sciences, Technology, and Innovation, Kiambu, Kenya; ^2^Department of Computing and Information Technology, Jomo Kenyatta University of Agriculture and Technology, Kiambu, Kenya; ^3^Department of Economics and Finance, School of Business and Public Administration, University of The Gambia, Serekunda, Gambia; ^4^Department of Statistics and Actuarial Sciences, Jomo Kenyatta University of Agriculture and Technology, Kiambu, Kenya

**Keywords:** economic indicators, gross domestic product, LSTM, prediction, remittances, RNN, survey, The Gambia

## Abstract

Insights into the magnitude and performance of an economy are crucial, with the growth rate of real GDP frequently used as a key indicator of economic health, highlighting the importance of the Gross Domestic Product (GDP). Additionally, remittances have drawn considerable global interest in recent years, particularly in The Gambia. This study introduces an innovative model, a hybrid of recurrent neural network and long-short-term memory (RNN-LSTM), to predict GDP growth based on remittance inflows in The Gambia. The model integrates data sourced both from the World Bank Development Indicators and the Central Bank of The Gambia (1966–2022). Pearson’s correlation was applied to detect and choose the variables that exhibit the strongest relationship with GDP and remittances. Furthermore, a parameter transfer learning technique was employed to enhance the model’s predictive accuracy. The hyperparameters of the model were fine-tuned through a random search process, and its effectiveness was assessed using RMSE, MAE, MAPE, and R^2^ metrics. The research results show, first, that it has good generalization capacity, with stable applicability in predicting GDP growth based on remittance inflows. Second, as compared to standalone models the suggested model surpassed in term of predicting accuracy attained the highest R^2^ score of 91.285%. Third, the predicted outcomes further demonstrated a strong and positive relationship between remittances and short-term economic growth. This paper addresses a critical research gap by employing artificial intelligence (AI) techniques to forecast GDP based on remittance inflows.

## 1 Introduction

One of the main macroeconomic indicators used to assess the economic performance of a nation or area is the growth rate of real GDP. The next economic goals and financial oversight plans will largely depend on this key metric, which highlights the significance of GDP (Gotz and Knetsch, 2019). Additionally, GDP offers valuable insights into the economic conditions at an individual level (Sa’adah and Wibowo, 2020). As economies undergo rapid changes, predicting GDP growth has emerged as a prominent area of study. These forecasts rely on complex models that often incorporate numerous variables, each requiring either a data driven parameter estimation or allocation based on assumptions. Example of such variables include financial indicators (e.g., interest rates, fiscal policies, public debt), socio-economic indicators (e.g., employment, labor productivity, demographic trends, education levels) and global trade indicators (e.g., trad openness, commodity price, exchange rates, remittances). While these multi-variables model creates the impression of considering all factors influencing economic development, they also aim to deliver accurate prediction (Tacchella et al., 2018).

GDP prediction has traditionally relied on statistical techniques obtained from economic enumeration, such as trend forecasting, regression modeling, and sequential data analysis (De Cola and Mongelli, 2018). However, these conventional approaches have limitations. Trend forecasting techniques based on empirical research are often demanding and prolonged, and the results from economic censuses can lag real-world developments. A major practical hurdle arises because time-series data such as GDP and remittances often deviate from the idealized assumptions of traditional time-series analysis and causal discovery methods. These assumptions typically presupposed stationarity, implying that system dynamics remain unchanged over time. However, real-world data exhibit seasonal fluctuations, abrupt shifts, and other complexities (Shams et al., 2024). For example, variations in remittance amounts during different periods challenge the assumption of stationarity. GDP is affected by a broad range of external elements, such as economic development levels, public policy priorities, local climate, and average income. Moreover, it has complex characteristics like volatility, feedback loops, and periodicity, making traditional prediction methods challenging.

In the age of big data, there are alternative approaches to GDP prediction, such as involving incorporating data-driven model and Artificial Intelligence (AI) algorithms. These models help in identifying and selecting relevant influencing features, leading to the construction of more robust GDP prediction models (Wu et al., 2021). Numerous AI and machine learning techniques have been employed to predict critical economic measures, such as GDP growth, Consumer Price Indices (CPI), unemployment rates, energy consumption, export, and interest rates. These approaches include Artificial Neural Networks (ANNs), Support Vector Regression (SVR) among others (Cicceri et al., 2020). Given the constraints of traditional machine learning techniques, the research community has increasingly turned to transfer learning. Conventional models like RNN, LSTM, CNN, GRU, and SVM require extensive training from the ground up, which is both computationally intensive and data-hungry to achieve optimal performance (Gu et al., 2021). These models also follow an isolated training approach, where each model is trained independently for a specific task without leveraging prior knowledge. To address these limitations, researchers are now adopting transfer learning, although its application in time series prediction remains limited (Nguyen and Yoon, 2019).

Moreover, remittance inflows play a vital role in stabilizing country’s economies, particularly in The Gambia by providing a significant source of foreign currency for imports. Given that, a large share of the goods used in The Gambia are imported, foreign currency is essential for both importers and the government (Ceesay et al., 2019). Currently, remittance is a major source of foreign currency, alongside Foreign Direct Investment (FDI), Official Development Assistance (ODA), and export of key agricultural products like groundnuts, cotton, and animal skins. Additionally, tourism contributes to foreign currency inflows, although its impact is subject to seasonal variations. In recent years, the migration of young, able-bodied individuals to more developed countries have also affected the labor force in The Gambia. Despite these challenges, remittance inflows have risen significantly, helping to stabilize foreign currency reserves and, by extension, maintains the prices of essential food items. Diaspora remittances is individual transfers from migrant workers residing abroad for over a year has contributed significantly to economic dynamics (UNDP, 2020; Adewale et al., 2021; Ceesay et al., 2019).

**Figure 1:**
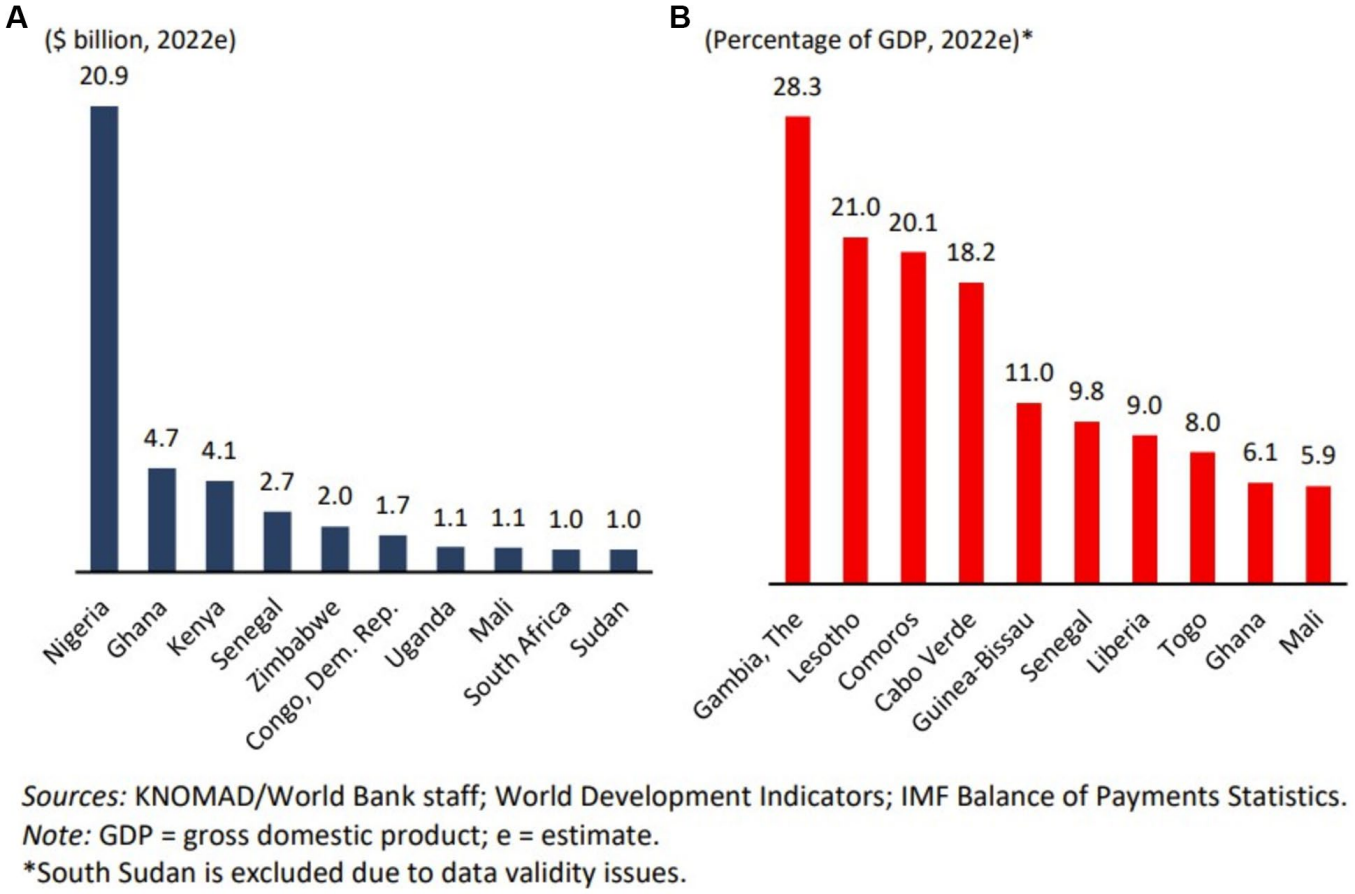
Top remittances recipients in the sub-Africa region, 2022e.

In 2022, several sub-Saharan countries stood out as significant recipients of remittances in US dollars. Nigeria, Ghana, Kenya, and Senegal led the pack (Figure 1A). Meanwhile, other countries, including The Gambia, Lesotho, Comoros, and Cape Verde, were heavily dependent on remittances as a share of their GDP (Figure 1B). In recent years, the top ten recipients have remained relatively stable, except for The Gambia, which experienced notable shifts due to significant political and economic changes (Ratha et al., 2022). The Gambia witnessed a remarkable surge in formal remittance inflows, rising by 200% over 5 years—increasing from US$278 million in 2018 to US$773 million in 2021, and reaching US$737.12 million in Ibenyenwa et al. (2023). These inflows accounted for 32.11% of the normal GDP, representing a 3.46% increase compared to the same period in 2022. According to the Central Bank of The Gambia, the remittance inflows in 2024 reached US$776 million. These inflows accounted for 31.5% of the normal GDP, marking a 4% increase compared to the same period in Ibenyenwa et al. (2023), which represents a notable increase of US$28.8 million compared to Ibenyenwa et al. (2023), GBoS (2022), and The Central Bank of The Gambia (2022).

In 2021, remittances accounted for a substantial 63% of the nation’s GDP, positioning The Gambia as one of the leading nations globally, relying on remittances. Despite this, Gambians face high costs associated with sending and receiving money within Africa (IFAD, 2022; The Central Bank of The Gambia, 2022). Like other developing countries, The Gambia receives remittances through both formal and informal channels. Formal remittances are handled by money transfer services such as MoneyGram, Western Union, and Ria, which work in partnership with commercial banks. These banks, with agents across the country, help distribute remittances to emigrants’ families. Data on official remittances is collected by financial institutions and submitted to the Central Bank on a weekly or monthly schedule. Conversely, informal remittances, managed by local agents, are not documented, making them challenging to track. This issue is particularly prominent among Gambians living in Russia. In our survey, respondents from Russia reported difficulties in sending money to their families in The Gambia due to frequent SWIFT network disruptions. As result, they avoid using formal channels like mobile money, online banking or money transfer apps. Instead, they rely on local agents for informal transfers, which are not officially recorded. A significant portion of remittances in The Gambia is used for household expenses, while a small share is invested in businesses, as indicated by the survey result in Section 3. Development experts believe that remittance inflows can play a vital role in reducing poverty and promoting economic growth (The Central Bank of The Gambia, 2022).

Our research contributes to existing literature in many ways. First, our study also delves into the relationship between remittance inflows and The Gambia’s economic growth (GDP growth) through a survey. The study also explores an important knowledge deficiency in the lack of specialized Artificial Intelligence (AI) and machine learning approaches, particularly ANN model, for predicting GDP growth- based remittance inflows in specific local contexts like The Gambia. Second, while existing studies analyzed the impact of remittance inflow on economic growth, they do not explore the predictions of GDP growth based on remittance inflows, especially using deep learning models based on transfer learning approach. Even though, many researchers have used neural networks for GDP/GDP growth predictions, but not many have applied an RNN-LSTM hybrid model for the prediction of GDP based on remittance inflows.

To our knowledge, this is the first study that looks at how to predict GDP growth based on remittance inflows. Therefore, the research gap exists due to the lack of ANN models specifically tailored for the dynamic and varied environments of countries like The Gambia, while efficiently managing non-linear and non-stationary data. Third, our study goes beyond traditional evaluation matrices by employing a comprehensive set that includes Root Mean Square Error (RMSE), Mean Absolute Error (MAE), Mean Percentage Absolute Error (MAPE), and the determination coefficient (R^2^) which was used in comparing the accuracy and stability of the suggested model with CNN, RNN and LSTM and we have shown that the suggested model is superior to the standalone models for predicting GDP growth based on remittance inflow. Finally, we also implemented a transfer learning approach called “parameter transfer approach” which leverages on parameter learned during pre-training from an initial domain and uses them to initialize or optimize a model for a new target domain by carefully selecting relevant features through Pearson Correlation (Wang et al., 2022; Mishra et al., 2020).

The rest of this paper is organized as follows. Section 2 reviews previous studies on remittance analysis and GDP forecasting methods. In Section 3, outlines the suggested framework. Section 4 covers the analysis and discussion of results. Lastly, Section 5 offers the conclusion and recommendations for the research.

## 2 Review of literature

Numerous studies have explored GDP forecasting with machine learning, offering diverse approaches and valuable perspectives. Predicting sequential data is essential in various fields, including meteorology, seismology, economics and finance. As we delved into time series prediction, we encountered various strategies: statistical forecasting, machine learning exploration, and the emergence of hybrid models (Han et al., 2024). Real-world time series data often exhibits non-linearity and non-stationarity, challenging conventional linear prediction methods. There are two prominent approaches in financial time series forecasting: conventional econometric methods and machine learning techniques (Li et al., 2019).

However, conventional econometric models with fixed parameters often struggle to analyze and intricate multi-dimensional and volatile financial data. In response to these limitations, neural networks have gained popularity for GDP prediction due to their capacity to derive insights from high-frequency raw data without requiring prior assumptions (Kogei, 2021). Furthermore, transfer learning enhances this approach by enabling the reuse of knowledge acquired from a pre-existing model, thus eliminating the need to construct a model entirely from the ground up. Typically, an already-trained model is developed using a sizable dataset, and the learned parameters from this model can be applied to a specialized neural network related to the same task. These modified models can be employed directly for predictions or further fine-tuned for comparable objectives, significantly reducing training time and minimizing generalization errors (Wu et al., 2022).

The study of Shams et al. (2024) introduces a novel Pearson Correlation-Long Short-Term Memory- Recurrent Neural Network (PC-LSTM-RNN) model for GDP prediction in urban areas, utilizing Pearson correlation for feature selection. The authors employed two datasets: a larger dataset with 227 instances and a smaller one containing 61 instances of historical GDP growth data from India. The proposed model utilizes parameter transfer to adapt features from the larger dataset to the smaller one, enhancing prediction accuracy. Results demonstrated the PC-LSTM-RNN’s strong predictive performance, with an R^2^ value of 99.99%, significantly outperforming other regression models by minimizing error rates and demonstrating high accuracy.

Cicceri et al. (2020) explores machine learning’s potential in forecasting economic recessions, using Italian GDP and related variables over 1995–2019. The authors compared traditional econometric techniques with the Nonlinear Autoregressive model with exogenous variables (NARX) and observe higher forecasting accuracy from the ML approach. The study highlighted the effectiveness of ML models in identifying recession patterns earlier than traditional methods, demonstrating that ML-based forecasting improves predictive accuracy, especially for economic downturns. In the study of Li et al. (2022), an ensemble decision framework for GDP prediction combines deep reinforcement learning (DQN) with predictors like GRU, TCN, and DBN models. Experimental cases in China showed that this ensemble approach outperforms traditional models by more than 10%, with MAPE values under 4.2%, demonstrating high prediction accuracy. This framework’s adaptability to different regional datasets presents a promising avenue for enhancing GDP forecasting accuracy and supporting economic policy planning.

Lai (2022) did a comparative study of different neural networks in predicting GDP. This study evaluates the effectiveness of three neural network models in predicting GDP, using data from Sichuan province between 1992 and 2020. The models compared include the genetic algorithm-back-propagation neural network, the particle swarm optimization (PSO)-Elman neural network, and the bat algorithm-long short- term memory (LSTM) model. Results showed that the PSO-Elman neural network outperformed the other models, with the lowest error rates, indicating its superior predictive capability for GDP. Zhang et al. (2022) research highlights the reliability of neural networks for economic forecasting. This research utilizes a Radial Basis Function Neural Network with Genetic Algorithm (RBFNN-GA) to forecast GDP for Shandong, emphasizing the interplay of inflation, unemployment, and growth. The genetic algorithm optimizes the network’s smoothing and central parameters, yielding accurate predictions that support economic decision-making. Comparative analysis shows that RBFNN-GA effectively captures nonlinear relationships within economic data, suggesting its applicability for macroeconomic forecasting Canadian GDP growth using XGBoost. Qureshi et al. (2020) explored the application of XGBoost, a machine learning algorithm, to forecast Canadian GDP growth using both Google Trends and official data. Employing a two- step feature selection approach, the study leverages Extreme Gradient Boosting combined with Principal Component Analysis (PCA) to improve prediction accuracy. The results demonstrate that XGBoost offers better forecasting performance compared to autoregressive and other boosting algorithms. Additionally, Google Trends data proved to be a valuable alternative when official data was unavailable, enhancing early predictions of Canadian GDP.

Velidi (2022) focuses on predicting the global GDP growth rate using multiple machine learning algorithms. By examining key variables that influence GDP across different countries, the study offers insights into the factors that drive economic performance. The use of visual diagrams aids in comparing the GDP growth rates of various countries, highlighting both high and low performance. The research underscores the importance of machine learning in understanding and forecasting global economic trends. The research presented in Longo et al. (2022) proposed an ensemble learning method to predict U.S. GDP growth by combining a Recurrent Neural Network (RNN) with a Dynamic Factor Model-Generalized Autoregressive Score (DFM-GAS) model. This approach integrates multiple economic indicators, including time-variant variables. The ensemble model performed well in forecasting short-term GDP, particularly during periods of economic instability, such as after the 2008–09 global financial crisis. The findings showed that the neural network ensemble significantly improves predictive accuracy compared to individual models.

In study of Muchisha et al. (2021), various machine learning (ML) models were developed to nowcast Indonesia’s GDP growth, using 18 macroeconomic and financial indicators. Six ML algorithms, including Random Forest, LASSO, Ridge, Elastic Net, Neural Networks, and Support Vector Machines, were assessed based on their accuracy from 2013 to 2019. Random Forest emerged as the best-performing model. Additionally, combining forecasts using LASSO regression with selected models further improved accuracy. The results underscore the utility of ML techniques in real-time economic forecasting, outperforming traditional autoregressive models. Blazsek et al. (2023) proposes a multivariate dynamic conditional score model, t-QVARMA, which integrates I (0) and I (1) components for GDP growth prediction alongside inflation and interest rates. The model outperforms the Gaussian-VAR counterpart in accuracy, especially in capturing short- and long-run economic impacts. Applied to U.S. data from 1954 to 2020, t-QVARMA demonstrates superior statistical performance and predictive accuracy, offering a robust method for analyzing economic relationships in GDP forecasting.

Han et al. (2024) presented an innovative framework that integrates signal processing techniques with advanced neural network architectures. Their methodology employs GARCH to capture volatility, CEEMDAN for decomposing data, and GCN for effective learning. The researchers evaluated their model across multiple datasets, demonstrating superior performance compared to traditional methods in terms of precision and robustness. In the study of Ibenyenwa et al. (2023) explored the effect of diaspora remittances on the Human Development Index (HDI) using data from 2002 to 2022. They assessed how foreign direct investment (FDI), per capita income, and remittances influenced HDI, though none had a significant impact. In their recommendations, they suggested that policymakers optimize remittance channels and promote FDI to strengthen the role of remittances in enhancing HDI.

Ceesay et al. (2019) studied personal remittances’ impact on The Gambian economy using dataset spanning from 2003 to 2017. They used the vector error correction model (VECM) method in their analysis. They found that personal remittances positively and significantly affect economic growth, increase foreign exchange, and reduce poverty. The research conducted in Zhu et al. (2024) proposed an innovative composite neural network framework, integrating predictive analytics and enhanced data preprocessing techniques to forecast stock prices. This hybrid model incorporates CEEMDAN-based preprocessing, LSTM, and convolutional layers. The model showed significant performance improvements, with a 45.33% reduction in MAE, a 43.44% decrease in RMSE, a 45.01% reduction in MAPE, and a 3.90% rise in R^2^.

A type of artificial neural network (ANN), known as the long short-term memory (LSTM) model, is particularly effective in handling economic time series data. In the study presented by Hopp (2022), the author analyzed the performance and characteristics of this architecture against the Dynamic Factor Model (DFM), a widely used approach in economic nowcasting. The findings revealed that LSTMs surpass DFMs in predicting three key indicators - worldwide merchandize export values, export quantities, and international services exports. The study of Tolo (2020), focused on predicting systemic financial crises 1–5 years ahead. He used the LSTM and Gated Recurrent Unit (GRU) neural nets which significantly improved predictions using historical macroeconomic data spanning from 1870 to 2016.

Chu and Qureshi (2023) assessed the performance of various machine learning and deep learning models for U.S. GDP growth forecasting. Examining the efficacy of density-based methods (e.g., bagging, boosting) and parsimonious models with high-frequency predictors, findings indicate that density-based methods outperform sparsity-based methods in short-horizon forecasting and that using a large set of predictors yield better results. For long-term forecasts, models with high-frequency predictors performed better, highlighting the importance of predictor selection in economic forecasting. Ensemble ML methods consistently surpassed other models, emphasizing their value in GDP prediction. Agu et al. (2022) study applies machine learning methods, including Principal Component Regression (PCR), Ridge Regression, Lasso Regression, and Ordinary Least Squares (OLS), to predict GDP based on macroeconomic indicators. With PCR achieving 89% accuracy, the study identifies key macroeconomic factors affecting GDP. The use of ML regularization methods over traditional statistical techniques allows more precise GDP predictions and emphasizes the impact of specific macroeconomic indicators, offering new insights into economic growth determinants.

## 3 Survey result

This section presents the findings of the survey. Remittances serve a crucial role in providing economic support to The Gambian families, with a large portion of the population relying on funds sent by relatives living abroad. The reliance on remittances is particularly evident in rural areas, where seasonal farming is the primary source of livelihood. The survey results provide a comprehensive understanding of the remittances patterns in The Gambia, including the countries where family members are located, the frequency, amounts, and methods employed for receiving money and main use of the remittances received.

### 3.1 Gambians living abroad and their locations

**Figure 2:**
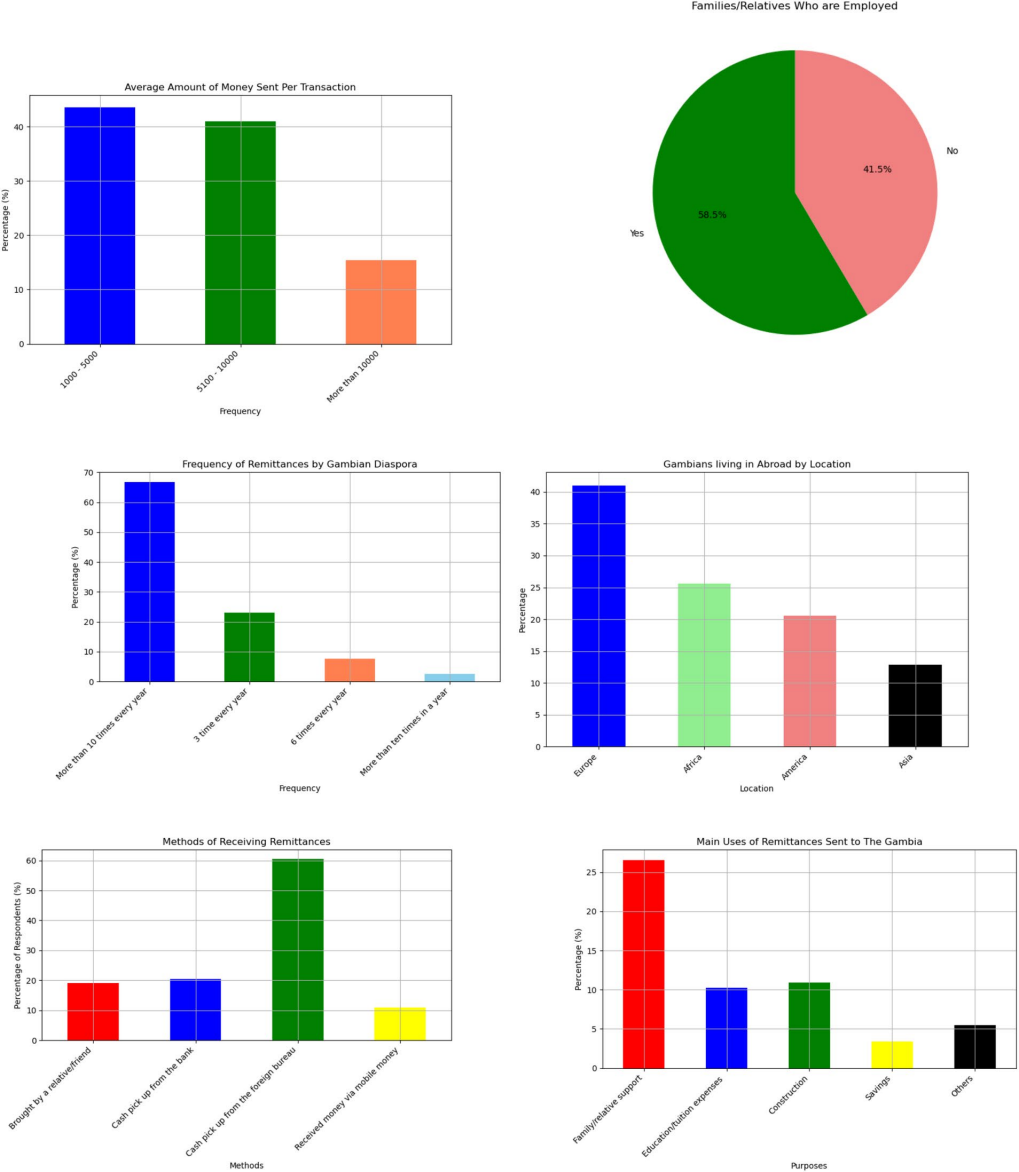
Survey results showcasing various aspects of remittance patterns, including average remittance amount sent, employment status, frequency of sending, location, methods of receiving, and purposes of receiving remittances.

Figure 2 shows that 63% of respondents have family members residing in Europe, and almost 25% have relatives based in the United States. Less than 10% have relatives living in Asia, and 2% have family in other parts of Africa. Nearly three-quarter of Gambians with relatives in Europe have family members residing in Spain, Italy, or Germany. This high concentration is primarily attributed to irregular migration through the so-called “back way.”

### 3.2 Sample profile

As shown in Figure 2, 58.5% of respondents are employed, while 41.5% are not. Table 1 illustrates the total sample of our survey. Most remittance recipients in rural areas work as seasonal farmers and gardeners, typically, in their late 50s. Women tend to grow vegetables in their gardens, while men, who engage in farming, rely heavily on rainfall for their crops. Youths are uncommon in rural areas, with nearly 90% of young people having undertaken risky journeys to Europe in search of better opportunities (Ceesay et al., 2019). According to the World Bank, almost 10% of The Gambia’s 2.7 million population have migrated, with the majority being young people from rural areas.

**Table 1:** Example profile.

Gender	Figure	Portion %
Male	144	75
Female	50	25

### 3.3 Frequency of remittance sent by Gambians in diaspora

Figure 2 shows that over 60% of Gambians in the diaspora who send money from abroad to The Gambia do so more than 10 times a year. This percentage is significant as it highlights the strong financial support that The Gambian diaspora provides to their home country. Regular remittances are crucial for many families in The Gambia, helping to cover essential expenses such as education, healthcare, and daily living costs. Additionally, this steady flow of funds contributes to the overall economic stability and growth of the country, showcasing the vital role that the diaspora plays in the nation’s development. Gambians living abroad send hundreds of millions of dollars annually in remittances, as reported by the World Bank (2019) and Ratha et al. (2022). These remittances make up a quarter of the small country’s economy, the highest share of any nation in Africa.

### 3.4 Average amount of money sent per transaction

On average, the amount of money sent to families or relatives in The Gambia per transaction is unlikely to exceed GMD 10,000 (US$142.86) according to our survey as shown in Figure 2. The most common range is between GMD 1,000 and GMD 50,000 per transaction.

### 3.5 Methods of receiving money from abroad

Survey results in Figure 2 indicate that over half of Gambians receiving remittances collect their money from foreign exchange bureaus, with 60% of respondents using this method. Another 20% pick up the money directly from a bank, 17% receive it from a relative or friend, and 5% use mobile money services.

### 3.6 Main uses of remittances received by families/relatives in Gambia

Most remittances are used to cover household costs, including essentials like food, water, and electricity Ratha et al. (2022) and Ceesay et al. (2019). More than half of respondents rely on remittances for daily sustenance, with some stating that they are entirely dependent on these funds for survival as shown in Figure 2.

Approximately, over 60% of the remittances received are used for family needs, while <20% go toward education or tuition fees. Only 7% of remittances are saved, and <10% are invested in businesses. A small portion, 11%, is used for property purchases or construction. The relatively small amounts directed toward savings and investment are because most remittances are intended for immediate consumption, leaving little surplus after meeting basic needs.

The survey results provide valuable context that supports the model’s predictive capacity. Specifically, they highlight how remittance inflows are predominantly allocated to immediate family needs, such as food, water, and electricity, with minimal portions saved (7%) or invested in businesses (<10%). This pattern underscores the role of remittances as a key driver of short-term economic activity, which aligns with the model’s focus on predicting GDP based on remittance inflows. The RNN-LSTM hybrid model’s high predictive accuracy (R^2^) validates its ability to capture the relationship between remittance inflows and GDP. The survey findings further substantiate this by demonstrating that remittances have a significant impact on consumption, a major component of GDP. This correlation between remittance driven consumption and economic growth provides empirical support for the model’s predictions. Additionally, the survey underscores the short-term nature of remittance-driven economic boosts, which the hybrid model effectively captures. By leveraging transfer learning and hybrid architecture, the model outperforms CNN, RNN, and LSTM models, demonstrating superior generalization and stability in forecasting GDP—a key finding that is consistent with the economic behaviors observed in the survey data.

## 4 Materials and methods

This section offers a comprehensive description of the suggested hybrid framework (RNN-LSTM). First, the dataset undergoes preprocessing, where we applied mean imputation and data normalization (Tarek et al., 2023). Furthermore, to improve the quality of the raw data and prepare it for modeling, this study employs mean imputation and data normalization techniques.

### 4.1 Data description

Remittance inflow was the primary factor identified as influencing economic growth (GDP per capita) in this study. To ensure a comprehensive analysis, we incorporated control variables such as FDI, Inflation, Exchange Rate, and Official Development Assistance (ODA), among others. For training the hybrid base model (Model A), we utilized socio-economic indicators in Dataset A, which were sourced from the World Bank Development Indicators database and Central Bank of The Gambia. Given that the data consisted of a mix of yearly and monthly entries, a preprocessing step was undertaken to align the temporal resolution of all variables. Specifically, yearly data were converted into monthly equivalents using interpolation techniques to ensure consistency with the monthly data. This transformation was critical for preserving temporal dependencies and optimizing the performance of the time-series model. Following the feature extraction process using Pearson Correlation, only the most relevant socio-economic indicators were retained and incorporated into Dataset B, which contains specific features. Dataset B was utilized to refine the predictive accuracy of the model, particularly for forecasting GDP growth based on remittance inflows in The Gambia. This approach allowed us to leverage the unique attributes of each dataset while maintaining robust predictive capabilities. Table 2 describes the key features of the datasets.

**Table 2:** Summary of the features of the dataset utilized.

Feature	Description
Date	The specific year for which the data is recorded
Country	The Gambia as the country
GBM	Country code, Th Gambia
GDP (per capita)	Adjusted gross domestic product per person, accounting for inflation to 2015 US dollars.
FDI	Net investment inflows to acquire management interest in enterprises outside the investor’s country, as a percentage of GDP.
Inflation	Annual percentage change in the GDP deflator, measuring price inflation.
Exchange rate	The exchange rate for the local currency relative to the US dollar during a specific timeframe.
Remittances	Total personal remittances received from abroad, as a percentage of GDP.
Net migration	Net number of people entering or leaving the country.
Population	Total number of people living in the country
Trade openness	Average of export and import percentages.
Human capital	the collective skills, knowledge, experience, and health levels of a population, which contribute to productivity and economic growth
ODA	Amount of aid received by The Gambia.
General	Government spending on final goods and services.
government	

### 4.2 Modeling

#### 4.2.1 Pearson correlation

The Pearson Correlation (PC) coefficient serves as a commonly employed metric for evaluating the relationship between two datasets and demonstrating the strength of their correlation (Benesty et al., 2008). To compute the Pearson correlation coefficient for two random variables ($X=x_{1},x_{2},\ldots \ldots .x_{n}$ and $Y=y_{1},y_{2},\ldots .y_{n}$) (where n represents the size of both X and Y) is shown below (Equation 1):


(1) $$r=\frac{\sum_{i=1}^{n}\left(x_{i}-\bar{x}\right)\left(y_{i}-\bar{y}\right)}{\sqrt{\sum_{i=1}^{n}{\left(x_{i}-\bar{x}\right)}^{2}\sum_{i=1}^{n}{\left(y-\bar{y}\right)}^{2}}}$$

Where $x_{i}$ and $y_{i}$ are the individual data points, $\bar{x}$ and $\bar{y}$ are the means of *x* and *y*, and *n* is the number of data points.

The strength of the correlation is regarded as greatest when the coefficient is equal to 1 or − 1. Specifically, the correlation can be understood as follows: values between 0.8 and 1 indicate a strong correlation; 0.6 to 0.8 denote a high correlation; 0.4 to 0.6 imply a moderate correlation; 0.2 to 0.4 suggest a weak correlation; and values from 0.0 to 0.2 signify a low or negligible correlation (Adler and Parmryd, 2010; Wu et al., 2021).

#### 4.2.2 Recurrent neural networks (RNN) model

Recurrent Neural Networks (RNNs) represent a category of artificial neural networks (ANNs) distinguished by feedback loops within their structure, allowing them to manage sequential data for tasks such as sequence recognition and forecasting (Bengio et al., 1994). RNNs are composed of multi- dimensional hidden states with non-linear dynamics (Sutskever et al., 2011). These hidden states act as the network’s memory, drawing upon their prior state Mikolov et al. (2014). RNNs can relate an input sequence to a corresponding output sequence at the present timestep and anticipate the subsequent timestep sequence (Salehinejad et al., 2017). They accept a sequence of vectors ($x_{1},x_{2},\ldots \ldots .x_{t}$) as input and produce another series ($h_{1},h_{2},\ldots \ldots .h_{t}$) that conveys the information from the input sequence at each step, as illustrated in Equation 1. RNNs are capable of processing sequences of different lengths by utilizing a recurrent hidden state that is contingent on the activation from the previous timestep (Huynh et al., 2017; Faru et al., 2023). Nevertheless, RNNs encounter difficulties in learning and training over extended temporal sequences due to the gradient problem, which can either excessively grow or shrink, impacting various layers and obstructing effective learning (Brownlee, 2018).

The update of the recurrent hidden state $h_{t}$ is executed as (Faru et al., 2023) (Equations 2, 3):


(2) $$h_{t}^{R}=f\left(WX_{t}+Uh_{\left(t-1\right)}^{R}+b\right)$$

(3) $$O_{t}^{R}=f\left(WX_{o}+Uh_{\left(t\right)}^{R}+b_{o}\right)$$

**Figure 3:**
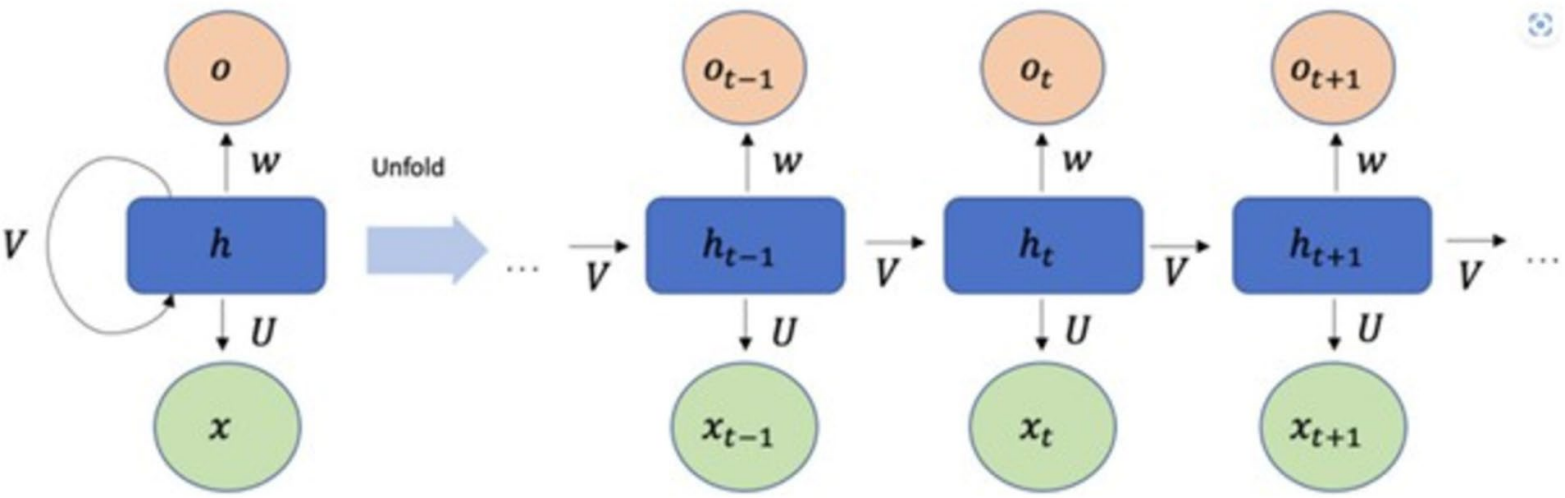
RNN model structure.

Where, f represents a non-linear function, typically utilizing ReLU. It acts as a hyper-parameter, similar to those in other neural networks, and $O_{t}^{R}$ is the predicted value derived from the recurrent neural network. At each iteration t, the hidden/current state $h_{t}^{R}$ depends on the input vector $x_{t}$ that the network gets at time t and the previous hidden state $h_{\left(t-1\right)}^{R}$ i.e. state at time (*t* − 1) and bias b. The weight matrices *W* and *U* serve as filters determining the significance of the current input and the former hidden state (Faru et al., 2023). The configuration of a typical RNN structure is depicted in Figure 3.

In essence, Recurrent Neural Networks (RNNs) utilize the identical network for every component in a sequence. RNNs retain and transmit pertinent information, allowing them to grasp temporal relationships that traditional neural networks are unable to comprehend.

#### 4.2.3 Long short-term memory (LSTM) model

**Figure 4:**
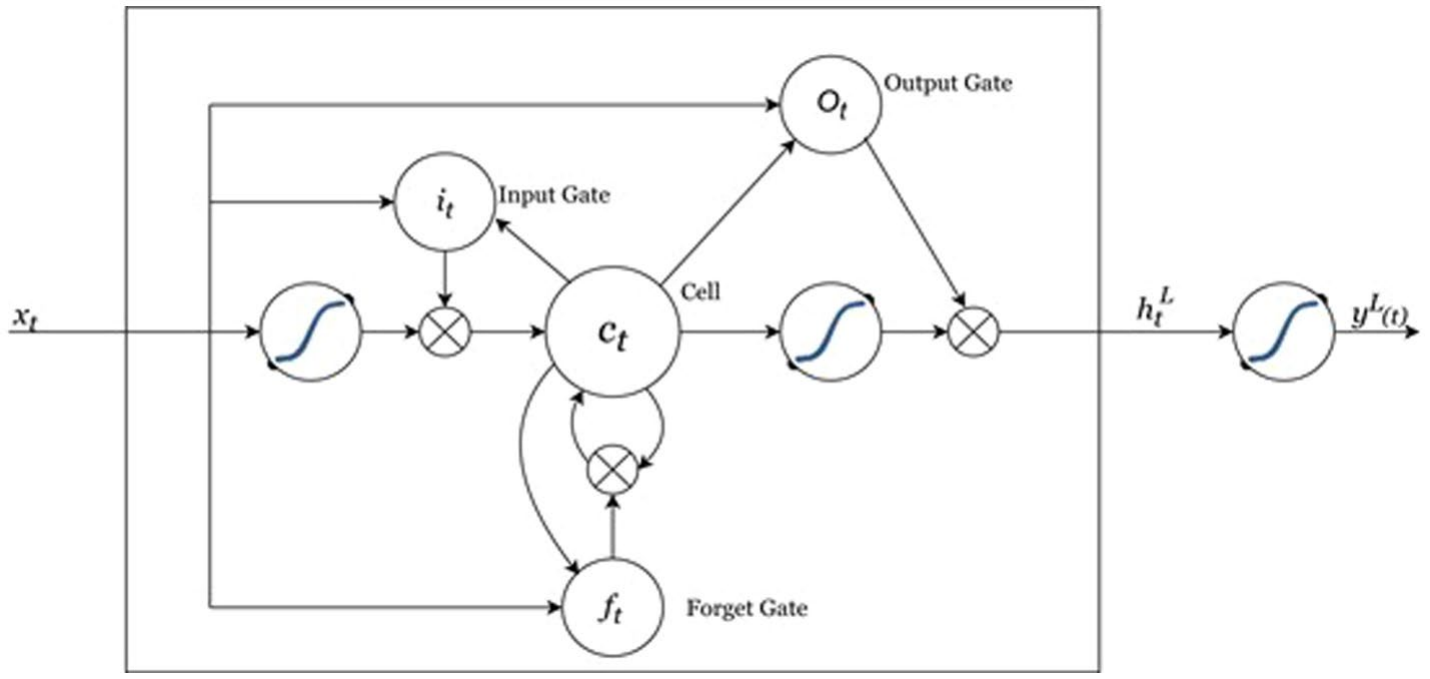
An LSTM cell architecture.

In 1997, Hochreiter and Schmidhuber introduced Long Short-Term Memory (LSTM), an advanced soft computing method developed to surpass the limitations of conventional RNNs, especially regarding their difficulty with long-term dependencies (Shahid et al., 2020). In 2015, Staudemeyer noted that RNNs presented a variant incorporating LSTM to alleviate issues related to vanishing and exploding gradients, by virtue of its competence in grasping both short- and long-term dependencies and its structure that addresses the vanishing gradient challenges commonly encountered in many RNN architectures. LSTM models are part of the deep recurrent neural networks family and are widely utilized in areas such as GDP forecasting and various time-series analyses (Salehinejad et al., 2017) due to their intrinsic memory capabilities and precision in making predictions (Lu et al., 2020). Consequently, they are well-suited for a range of sequential tasks. The structure of the LSTM memory cell includes three components: the forget gate, the input gate, and the output gate, as depicted in Figure 4. The equations presented below illustrate the mathematical functioning of an LSTM cell (Faru et al., 2023):


(4) $$i_{t}=\sigma \left(W_{\text{xi}}X_{t}+U_{ht}h_{\left(t-1\right)}^{L}+b_{t}\right)$$

(5) $$f_{t}=\sigma \left(W_{xf}X_{t}+U_{hf}h_{\left(t-1\right)}^{L}+b_{f}\right)$$

(6) $$o_{t}=\sigma \left(W_{xo}X_{t}+U_{ho}h_{\left(t-1\right)}^{L}+b_{o}\right)$$

(7) $$U=\tanh\left(W_{xu}X_{t}+U_{hu}h_{\left(t-1\right)}^{L}+b_{u}\right)$$

(8) $$c_{t}=f_{t}⊙C_{\left(t-1\right)}+i_{t}⊙U_{t}$$

(9) $$h_{t}^{L}=\tanh\left(c_{t}\right)⊙o_{t}$$

(10) $$y_{t}^{L}=\sigma \left(W_{o}h_{t}^{L}\right)$$

In the Equations 4–10 above, $i_{t}$ is input gate, $f_{t}$ is the forget gate, $o_{t}$ is the output gate $c_{t}$ is a cell state, $U_{t}$ is the activation function, $W_{\text{xi}}$ and $U_{hi}$ are weight matrices for input features and hidden state, respectively, $h_{t}^{L}$ is the LSTM hidden state, $X_{t}$ represents the input features at time step (t), ⊙ denotes elementwise multiplication, *σ* denotes Sigmiod activation operation, and $y_{t}^{L}$ is the predicted value. The weights *W* and biases *b* corresponds to the learned gates of the LSTM. The layout of a typical LSTM neural network is illustrated in Figure 4 (Faru et al., 2023).

Figure 4 depicts the connections between these LSTM blocks. In contrast to a conventional recurrent block that merely updates its state at each time interval, an LSTM has the ability to dynamically determine if it should keep or alter the present memory using its supplementary gates. In particular, the output gate manages the accessibility of the internal memory state, whereas the input gate decides which new data ought to be integrated. Additionally, the forget gate governs the degree to which the prior memory cell is discarded (Faru et al., 2023).

#### 4.2.4 RNN-LSTM model

**Figure 5:**
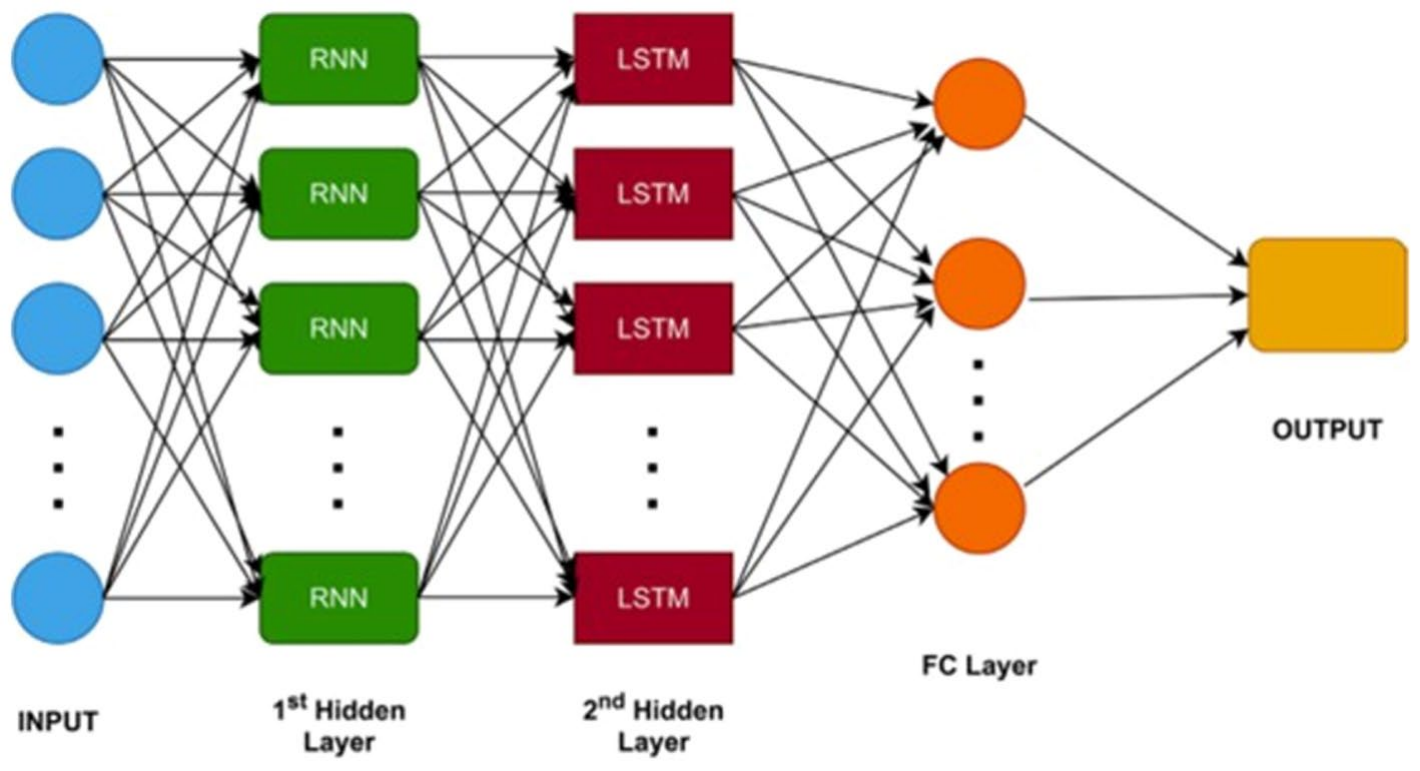
A foundational model architecture combining RNN and LSTM elements.

An RNN consists of neurons connected through weights, enabling the processing of inputs of varying sizes. RNNs excel at handling sequential data, such as remittance and GDP, by maintaining an internal state that encodes information about the observed timesteps. LSTMs, a specific type of RNN, enhance this by addressing the vanishing gradient problem and capturing long-range dependencies more effectively (Shams et al., 2024). We adopted a sequential input method to present the model attributes in a specific rank. The RNN-LSTM hybrid model integrates the advantages of both RNN and LSTM frameworks, effectively capturing temporal dependencies via this sequential format. This integration of memory retention methods from RNNs and LSTMs, combined with the sequential input approach, allows the model to more effectively identify dependencies within the data, leading to more accurate predictions. The architecture of the RNN- LSTM hybrid model is illustrated Figure 5 (Faru et al., 2023), which includes the input layer, RNN hidden layer, LSTM hidden layer, fully connected layer, and output layer. We implement three gates: Forget gate, Input gate, and Output gate. The primary reason for utilizing these networks is that predicting GDP growth is a regression task. Initially, data is input into the RNN, producing an output denoted as $O_{t}^{R}$ in Equation 11. For the final forecast $\hat{y_{t}}$, the RNN’s output is fed into the LSTM layer. The result from the RNN-LSTM hybrid model is mathematically derived as shown below (Faru et al., 2023) (Equations 12–15):


(11) $$i_{t}^{H}=\sigma \left(W_{\text{xi}}O_{t}^{R}+U_{hi}h_{\left(t-1\right)}^{L}+b_{i}\right)$$

(12) $$f_{t}^{H}=\sigma \left(W_{xf}O_{t}^{R}+U_{hf}h_{\left(t-1\right)}^{L}+b_{f}\right)$$

(13) $$o_{t}^{H}=\sigma \left(W_{xo}O_{t}^{R}+U_{ho}h_{\left(t-1\right)}^{L}+b_{o}\right)$$

(14) $$U_{t}^{H}=\tanh\left(W_{xu}O_{t}^{R}+U_{hu}h_{\left(t-1\right)}^{L}+b_{u}\right)$$

(15) $$c_{t}^{H}=f_{t}^{H}⊙C_{\left(t-1\right)}^{H}+i_{t}^{H}⊙U_{t}^{H}$$

(16) $$h_{t}^{H}=\tanh\left(c_{t}^{H}\right)⊙o_{t}^{H}$$

And finally, the predicted price $\hat{y_{t}}$ is given by Equation 17;


(17) $$\hat{y_{t}}=\sigma \left(W_{o}h_{t}^{H}\right)$$

where: $h_{t}^{H}$ represents the hidden state of the LSTM unit as defined by Equation 16. This state is derived by utilizing the tanh activation function on the revised cell state, followed by a multiplication with the output gate, $O_{t}^{H}$ is data is output from the RNN model $i_{t}^{H}$ is the input gate for the hybrid model at time step *t*.

According to Islam et al. (2020), these are advanced neural network models capable of achieving greater accuracy than regression-based predictions.

#### 4.2.5 Transfer learning

**Figure 6:**
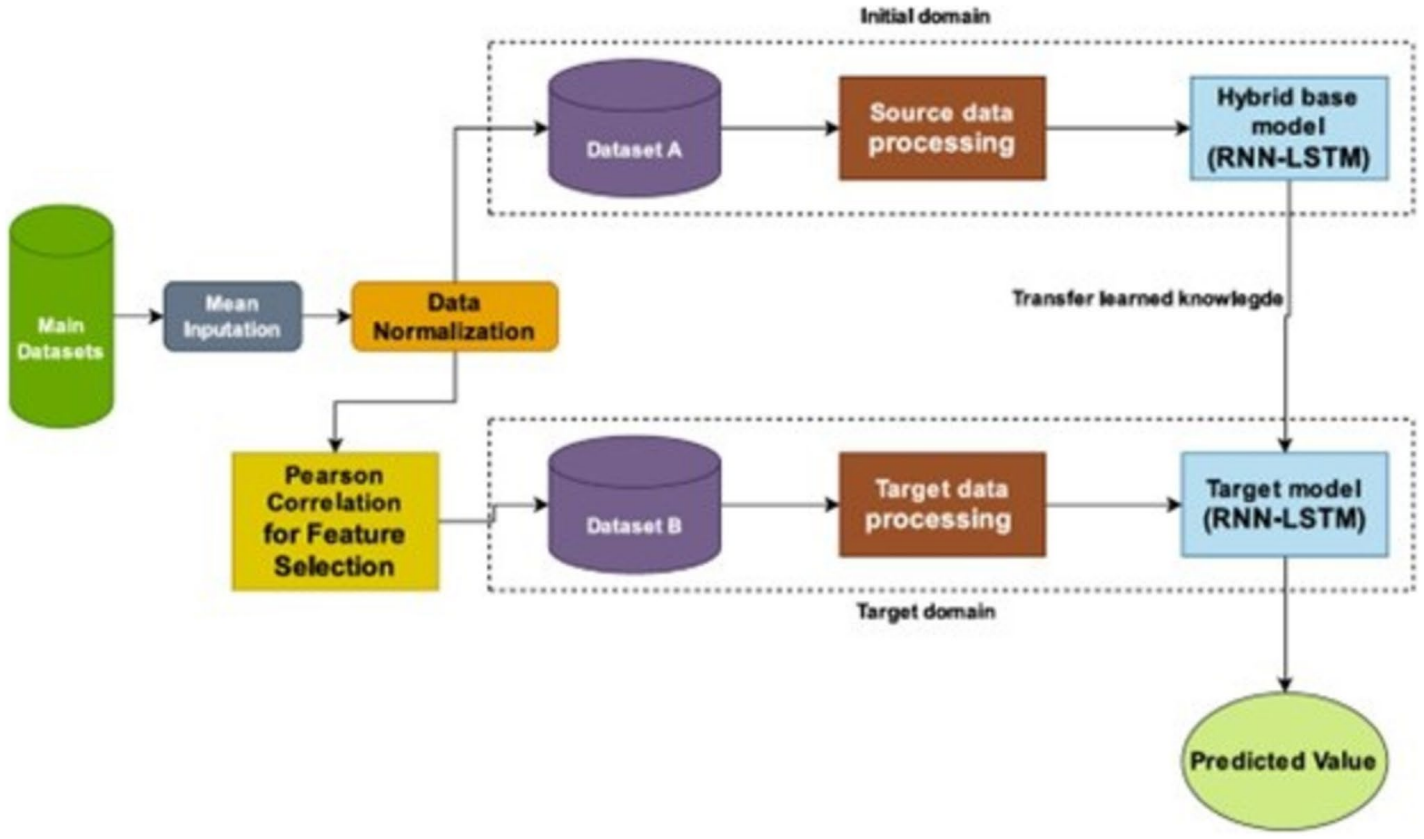
The overall framework of the suggested RNN-LSTM model: transfer learning structure.

The “parameter transfer approach” (Shams et al., 2024; Wang et al., 2022) adopted in this study entails utilizing the parameters obtained from the pre-training of a hybrid RNN-LSTM model on dataset A, which is large dataset comprises of socio-economic indicators, and transferring these parameters to either initialize or refine a model for GDP forecasting on dataset B which contain specific features. Dataset A is employed to train the initial model, enabling the model to discern the relationships between economic indicators, such as remittance, and GDP growth. The acquired parameters, encompassing weights and biases, are subsequently shifted to a new model (target model) intended for GDP forecasting on dataset B, which includes features chosen based on Pearson Correlation. In this methodology, features from dataset B that show a strong correlation with GDP per capita are combined with the pre-trained model. This combination aids in refining the target model, boosting its capacity to predict GDP growth based on remittance inflows by leveraging both the learned patterns from dataset A and the specific features of dataset B. The methodology integrates time-series analysis through the application of Recurrent Neural Networks (RNNs) and Long Short-Term Memory (LSTM) networks, merging the temporal information from both datasets. By conveying knowledge from dataset A to dataset B, this approach enhances the performance of the hybrid RNN-LSTM model, thereby increasing its predictive accuracy for GDP estimates. Utilizing advanced techniques like regularization and normalization, this process seeks to improve the model’s ability to capture intricate economic dynamics for GDP forecasting based on remittance inflows in The Gambia (Shams et al., 2024). Figure 6 illustrate how the transfer learning architecture using our suggested model for both dataset A (initial domain) and dataset B (target domain).

## 5 Experiment and results

The configuration of both the software and hardware used in the study experiment is presented in Table 3.

**Table 3:** Setup of an experimental environment.

Item	Parameter
Processor (CPU)	Core(TM) i7-7600U CPU@2.80GHz
Graphics Card (GPU)	Intel(R) HD Graphics 620
Memory (RAM)	16.00 GB
Programming language	Python 3.12.0
Deep learning framework	TensorFlow v2.16.1

### 5.1 Data preprocessing

#### 5.1.1 Mean imputation and feature engineering

To address missing data, two primary strategies are often employed (Donders et al., 2006): (1) imputation of missing values, or (2) discarding data samples containing missing entries. In the case of univariate imputation, methods such as mean or median imputation, forward and backward filling, and moving average imputation are commonly used (Van Buuren et al., 2006). Meanwhile, multivariate imputation often relies on KNN-based and regression-based methods. This study applies mean univariate imputation to handle missing values. As part of the data preprocessing, we handled missing values through imputation and then conducted feature engineering to create a new variable, Trade Openness. This variable was derived by combining the exports of goods and services and imports of goods and services variables. Specifically, Trade Openness was calculated by summing the values of exports and imports and dividing the result by two to capture the average trade activity. Trade openness, an indicator of economic integration, reflects a country’s degree of accessibility to foreign markets, which can impact GDP, economic growth, and overall trade balance. By incorporating this feature, we aim to enhance the model’s ability to capture economic dynamics influenced by international trade.

#### 5.1.2 Data splitting and normalization

The dataset was divided into three subsets: 75% was allocated for training, 25% for validation, and another 25% for testing. After splitting, we applied the MinMaxScaler function to scale the data, eliminating potential biases by standardizing all values within a range of [0, 1]. This function adjusted each column based on its minimum and maximum values, ensuring all data in a given column was scaled consistently across the dataset. We performed normalization after splitting to ensure both the training and test sets were scaled according to the training set’s parameters, aligning with the model’s focus on training data rather than test data.

Next, we reshaped the data into a 3D array format compatible with LSTM and RNN models in Keras, a deep learning library for Python that operates atop TensorFlow. The training dataset was structured as (505, 10, 14) and the testing dataset as (169, 10, 14), using the sliding window method. This structure represents the number of samples, time steps, and features in each dataset. Here, the 10-time steps indicate the count of prior observations used for predictions, and the 14 variables represent the economic indicators incorporated into the model. For both the training and test sets, we applied further normalization through feature scaling using min-max normalization. This scaling ensures consistency across features and enhances model performance by standardizing the data within a defined range.

### 5.2 Statistical analysis

**Figure 7:**
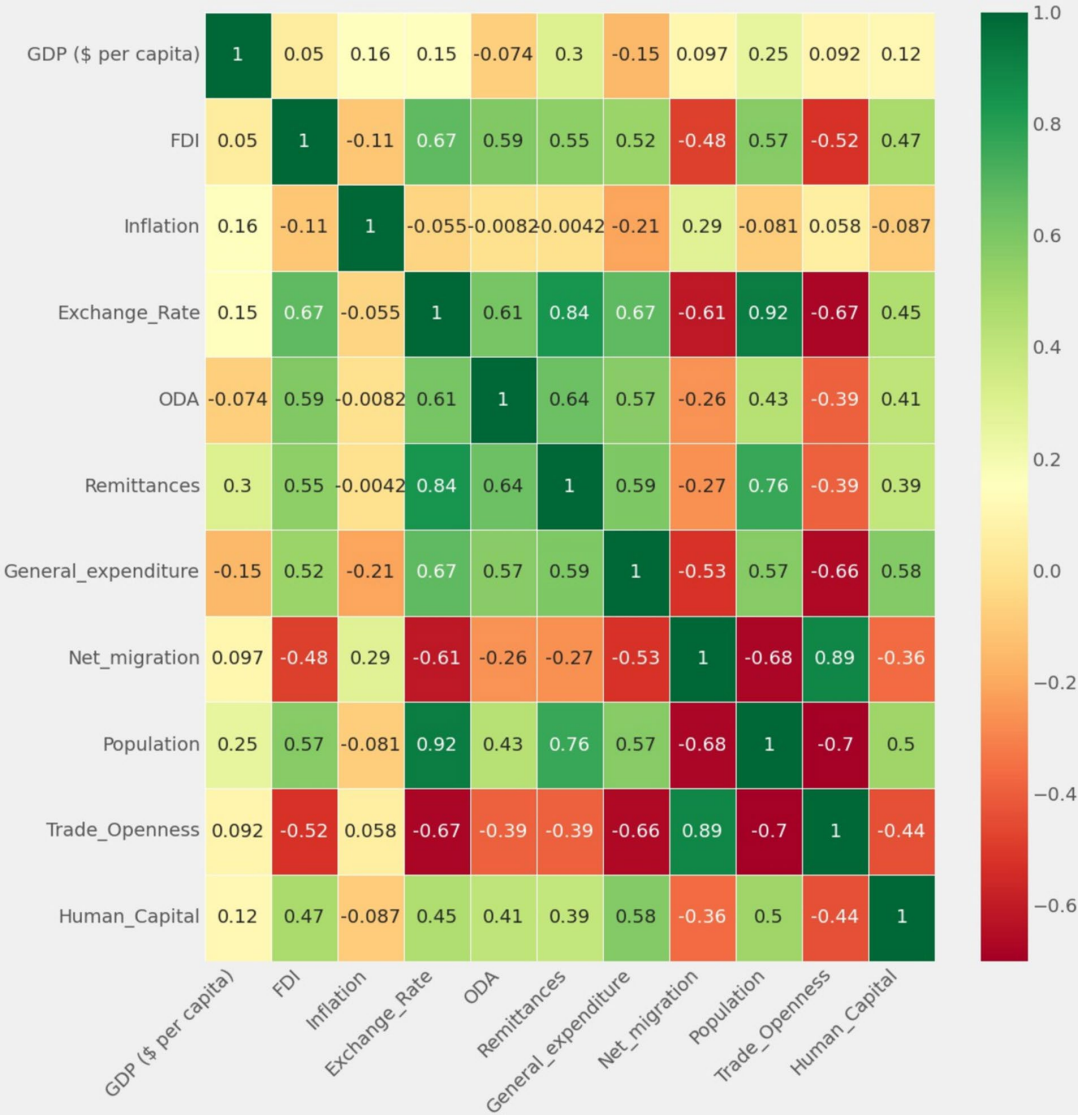
Feature analysis using a heatmap.

Figure 7 illustrates the heatmap analysis of the dataset’s features. The heatmap visually represents correlations and aids data visualization across all features. It analyzes the input features in relation to the target variable (GDP), uncovering relationships and patterns present in the dataset. Intensity variations in the heatmap indicate correlation strength and direction, providing nuanced insights into inter-dependencies. Notably, the diagonal exhibits a high positive correlation (color-coded green), while negative correlation (color-coded red) indicates minimal values.

The correlation analysis among the study variables reveals positive correlations between remittances, foreign direct investment (FDI), exchange rate, government expenditure, and trade openness. A weak positive correlation (correlation coefficient of 0.298796) exists between GDP per capita and remittances. Notably, there is no correlation between trade openness and net migration. While this suggests that remittances are often used for consumption rather than investment, it is essential to recognize that correlation does not imply causality, and this argument is highlighted in the survey results.

**Figure 8:**
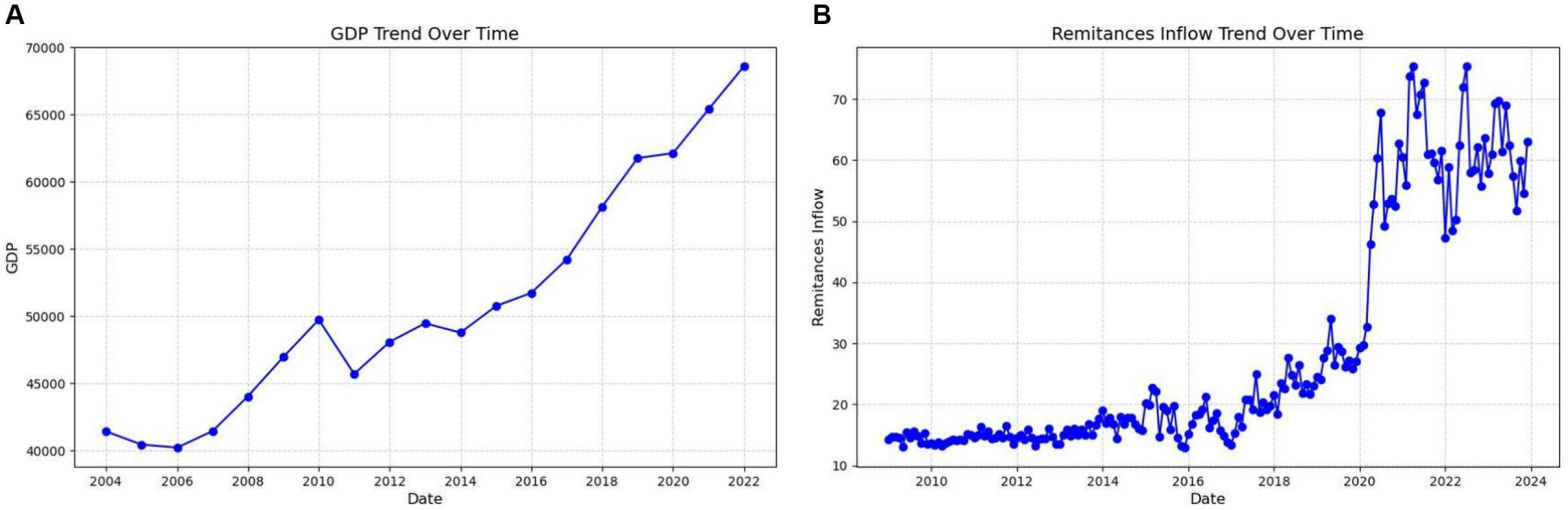
The left sub-figure **(A)** shows the GDP growth over time and the right sub-figure **(B)** shows the remittance trend over time.

As shown in Figure 8B, remittance inflow has changed significantly since 2020. In the 1990s and early 2000s, the inflow of remittances was relatively stable. However, this stability ended in the early 2000s as remittance inflows began to increase. Following 2020, remittance inflows experienced pronounced volatility, likely due to the economic impact of the COVID-19 pandemic, which was declared a Public.

Health Emergency of International Concern on 30 January 2020 by the World Health Organization. The sharp increase in remittance inflows during this period can be attributed to the global pandemic, which led to widespread job losses and economic hardship. Many families and relatives of migrant workers were laid off or faced severe financial difficulties. As a result, these families became highly dependent on diaspora remittances to support themselves and meet their basic needs. The surge in remittances during this period highlights the critical role of migrant workers in providing financial support to their home countries, especially during times of crisis. GDP growth has undergone significant changes since approximately 2007. From 2012 to today, The Gambia has experienced consistent increase in GDP growth, with only minor fluctuations along the way, as illustrated in Figure 8A.

Table 4 presents the correlation values between the predictors and the outcome variable. The correlation value of 0.299, as shown in Table 4, indicates that remittances have a weak or moderate positive correlation with GDP per capita. This would indicate a moderate relationship but confirms that remittances alone are not the only major driver in GDP growth. This aligns with the re-feedback findings of Ceesay et al. (2019), in which remittances tend to be used more for the immediate needs of family members (e. g., food, water, and electricity) and much less often for savings or investments. But because they are used mainly for consumption purposes and not so much to fund investments, the effect on GDP growth is expected to be very limited. The fact that remittances contribute more than 63% to the growth of the GDP of The Gambia, as reported by the Central Bank of The Gambia (World Bank, 2019), seems to contrast with the weaker correlation values between remittances and GDP per capita.

**Table 4:** The correlation indices of features associated with the target feature.

Feature	Correlation values
Remittances	0.299
Population	0.245
Inflation	0.156
Exchange Rate	0.146
Human Capital	0.117
General expenditure	−0.154

The Central Bank’s figures more likely measured the direct financial contribution of remittance to GDP showing the sheer amount of money flowing into the economy from abroad. The over 63% reflects the monetary value between the remittance in relation to the total GDP, demonstrating how much the remittances inflated the overall economic size. However, the correlation value (0.298796) from the Pearson.

Correlation measures the statistical relationship between remittances and GDP per capita. This value does not account for the volume of remittances but rather examines whether changes in remittances inflow directly leads to changes in GDP per capita growth.

The survey results show that most of the remittances are used for immediate family needs (e.g., food, water, electricity etc.) with only 7% is saved and <10% is invested in businesses. In this context, remittances serve as a key driver of consumption, sustaining a lot of families who might face poverty. So, this boosts short-term economic activity but not actually lead to long-term capital accumulation for improvements in productivity. The Gambia’s economy is highly dependent on consumption-based activities, and remittances are increasing this part of the economy. Therefore, the contribution to the growth of GDP of 63% from remittances reflects the essential role remittances play in keeping consumption and domestic demand buoyant. However, because this money is not importantly channeled into investment or development of human capital, its ability to drive sustained GDP per capita growth over time might be less pronounced, which is why the correlation value is not higher.

The Central Bank’s figure of remittances contributing more than 63% to GDP growth shows immediate financial benefits. However, the fact that remittances are mainly used to cover expenses of daily living limits their ability to create long-term economic change. The survey shows that only a small amount of the remittances (11%) is invested in property or construction, and even less is invested in businesses. The moderate Pearson correlation value reflects the direct linear relationship between remittance inflows and GDP. However, when remittances are analyzed in a multivariate framework, their critical role as a driver of GDP becomes evident. This underscores the importance of considering remittances within a broader economic context to fully understand their impact on GDP dynamics. Additionally, while remittances provide short-term economic stability, they do not contribute as much to long-term development indicators like GDP per capita, which explains the moderate correlation value from the Pearson correlation table. While remittances remain a significant predictor of GDP growth, the study also underscores the positive but moderate influence of population size on economic performance. Distinguishing these factors allows for a more nuanced understanding of their respective roles in shaping GDP growth.

### 5.3 Performance metrics

To showcase the robustness of the suggested hybrid RNN-LSTM model, four uncertainty analysis metrics are introduced: Root-Mean-Square Error (RMSE), Mean Absolute Error (MAE), Mean Absolute Percentage Error (MAPE), and the coefficient of determination (R^2^) as shown in equations below (Equations 18–21) (Hassan et al., 2022; Hossain et al., 2018).


(18) $$\text{RMSE}=\sqrt{\frac{1}{N}\sum_{t=1}^{N}{\left(\hat{y_{t}}-y_{t}\right)}^{2}}$$

(19) $$MAE=\frac{1}{N}\sum_{i=1}^{N}|\hat{y_{i}}-y_{t}|$$

(20) $$\text{MAPE}=\frac{1}{N}\sum_{i=1}^{N}|\frac{\hat{y_{i}}-y_{t}}{y_{t}}|$$

(21) $$R^{2}=1-\frac{\sum_{t=1}^{N}{\left(\hat{y_{t}}-y_{t}\right)}^{2}}{\sum_{t=1}^{N}{\left(y_{t}-\bar{y_{t}}\right)}^{2}}$$

Where *N* is the total number of data points, $y_{t}$ is the actual value, and $\hat{y_{t}}$ signifies the predicted value.

## 6 Discussion

**Figure 9:**
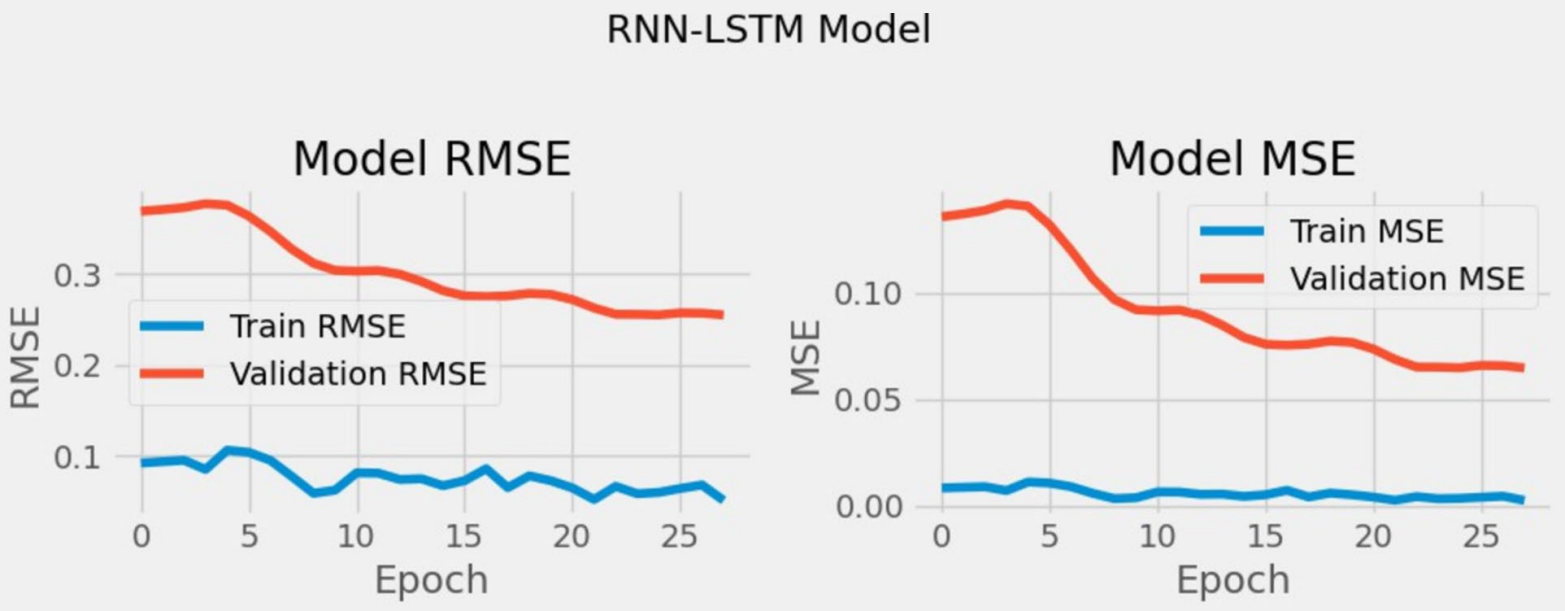
RMSE and loss prior to parameter optimization.

In section 6, we investigate the predictive capability and show the detail impact of each hyperparameter setting on RMSE, convergence, and model robustness of the RNN-LSTM hybrid model. We used the data shown in Table 2 to train and evaluate our models including CNN, RNN, LSTM and the hybrid RNN-LSTM model for the initial domain. We conducted training and validation of the foundational model for our hybrid RNN-LSTM architecture and the standalone models utilizing the dataset A, as illustrated in Table 5. From Table 5 it has been concluded that the RNN model performed much better than hybrid model, CNN and LSTM models in terms of prediction accuracy. We also validated our hybrid base model using the default parameters, employing RMSE and Loss (MSE) for evaluating the performance and the result is shown in Figure 9.

**Table 5:** Evaluation of model predictions.

Model	RMSE	MAP	MAPE	R^2^
CNN	0.114021	0.060726	0.062938	0.646915
RNN	0.099990	0.059039	0.087402	0.728466
LSTM	0.110370	0.070943	0.106847	0.669162
RNN-LSTM	0.110370	0.070943	0.106847	0.669162

To minimize the training and validation RMSE and loss, we explored various configurations of key hyperparameters (number of units, batch size, learning rate, dropout rate, and hidden layers) using a random search of our hybrid RNN-LSTM base model, as shown in Table 6. To efficiently explore the extensive search space, random search was employed as a method to identify the optimal hyperparameter settings.

**Table 6:** Hyperparameters.

Hyperparameter	Values
Count of layers	2–3
Number of neurons per layer	20–80
Batch size	16, 32, and 64
Number of epochs	20–100
Dropout rate	0.1–0.5

**Figure 10:**
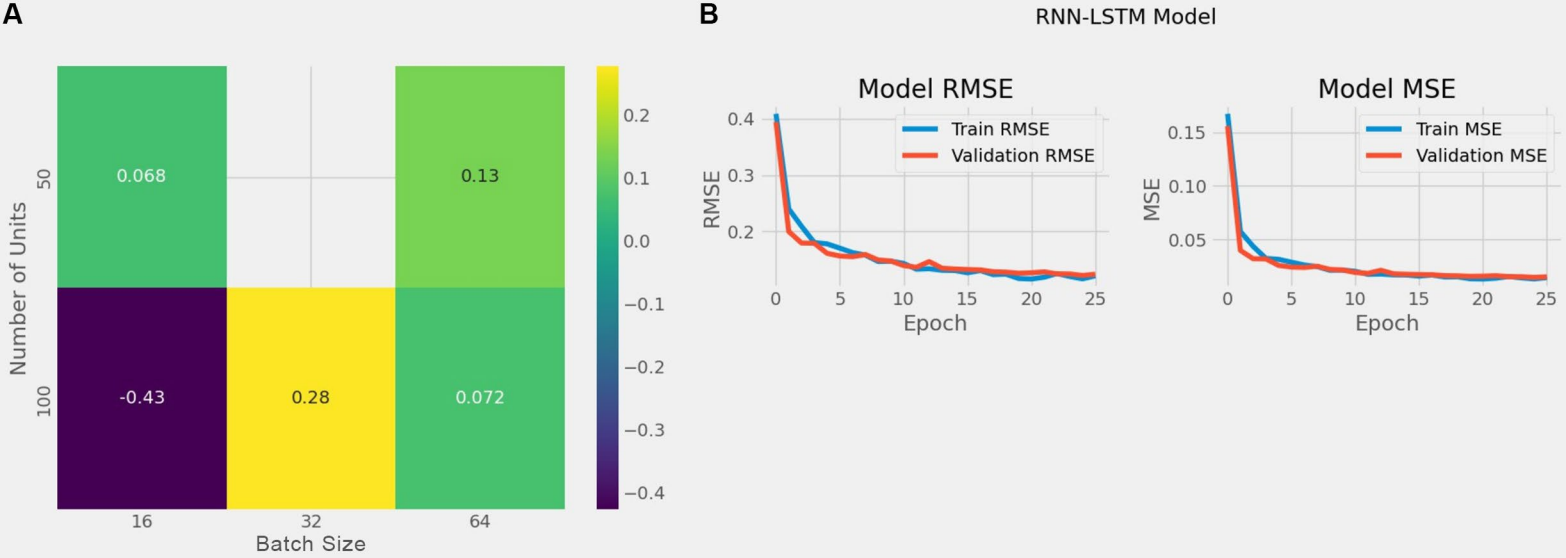
The left sub-figure **(A)** shows the heatmap illustrating RMSE values for combinations of units and batch sizes and the right sub-figure **(B)** shows RMSE and loss following parameter optimization.

The best combination of hyperparameters, which resulted in the lowest error for all our model, we found that a learning rate of 0.0001 provided the best RMSE performance, ensuring stable convergence and a smooth learning curve, while higher learning rates (e.g., 0.001 or above) led to faster initial learning but with more fluctuations in RMSE, indicating overfitting potential. For batch size, a setting of 16 yielded the lowest RMSE values across units, indicating better generalization due to more variability in gradient updates, whereas larger batch sizes (e.g., 64) showed faster training times but were prone to convergence issues, often resulting in higher RMSE scores due to underfitting. The chosen dropout rate of 0.2 provided optimal performance by ensuring regularization without excessive loss of information, while higher dropout rates (e.g., 0.3 or 0.4) resulted in slower convergence and sometimes higher RMSE scores, likely due to reduced capacity in the network. The best combination of hyperparameters is presented in Table 7 and the enhanced RMSE and Loss values for our hybrid model is illustrated in Figure 10.

**Table 7:** Optimal set of hyperparameters.

Hyperparameter	Values
Count of layers	3
Number of neurons per layer	80
Are the layers completely interconnected?	Yes
Size of batch	16
Learning rate	0.0001
Number of epochs	100
Dropout rate	0.2
optimizer	Adam
Time steps	10
Activation function	Relu
Loss function	Mean absolute error

To provide a clearer picture of the impact of different hyperparameter settings on RMSE, we generated a heatmap illustrating RMSE values for combinations of units and batch sizes as shown in Figure 10A. The heatmap results indicate that the best hyperparameters combination is 100 units with a batch size of 16, which showed the best overall performance with an RMSE of −0.43, suggesting effective generalization and minimized loss. Additionally, smaller batch sizes combined with 80 or 100 units consistently showed lower RMSE values, highlighting the importance of batch size and units in balancing the RNN-LSTM model’s capacity and training efficiency.

Following the training and validation of our hybrid foundational model, we moved the acquired weights and biases to the target model and we applied the weights and biases from our foundational hybrid model to the target model to predict GDP growth based on remittance inflows and subsequently trained it using dataset B on top of it, and this was done to ensure that there was no overfitting. To predict GDP growth based on remittance inflows in The Gambia, we used our dataset B which contains the most correlated economic indicator features with target feature after applying Pearson correlation. This approach employs a comprehensive methodology: selecting relevant attributes from dataset A and utilizing transfer learning through a parameter transfer technique with additional layers specifically designed for predicting GDP growth prediction based on remittance inflows. For training the new model, we fixed the upper third of the foundation model’s layers, added our dense layer, trained it in batches of 16, and completed 100 epochs. To address overfitting, we implemented drop-out rate regularization and early stopping techniques to identify the best model determined through cross-validation. To assess the robustness of the suggested hybrid model (RNN-LSTM) in predicting GDP growth based on remittance inflows, we used CNN, RNN and LSTM models for comparison purposes. The efficacy of the models was assessed using RMSE, MAE, MAPE, and R^2^ metrics. Table 8 illustrates the prediction outcomes of our models following parameter optimization and relevant feature selection.

**Table 8:** Evaluation of model predictions.

Model	RMSE	MAP	MAPE	R^2^
CNN	0.102230	0.075619	0.184059	0.823338
RNN	0.093976	0.059343	0.117500	0.850711
LSTM	0.083124	0.050257	0.100706	0.883200
RNN-LSTM	0.071802	0.055395	0.137087	0.912850

As shown in Table 8, the proposed hybrid model (RNN-LSTM) performed now much better than other models in terms of predicting accuracy and yields the best performance metrics, with values of 0.071802 for RMSE, 0.055395 for MAE, 0.137087 for MAPE, and 91.28% for R^2^. In contrast, the CNN model recorded the lowest values of 0.102230 for RMSE, 0.075619 for MAE, 0.184059 for MAPE, and 82.33% for R^2^. The RNN model exhibited RMSE, MAE, MAPE, and R^2^ values of 0.093976, 0.059343, 0.117500, and 85.07%, respectively. Lastly, the LSTM model achieved values of 0.083124 for RMSE, 0.050257 for MAE, 0.100706 for MAPE, and 88.32% for R^2^.

**Figure 11:**
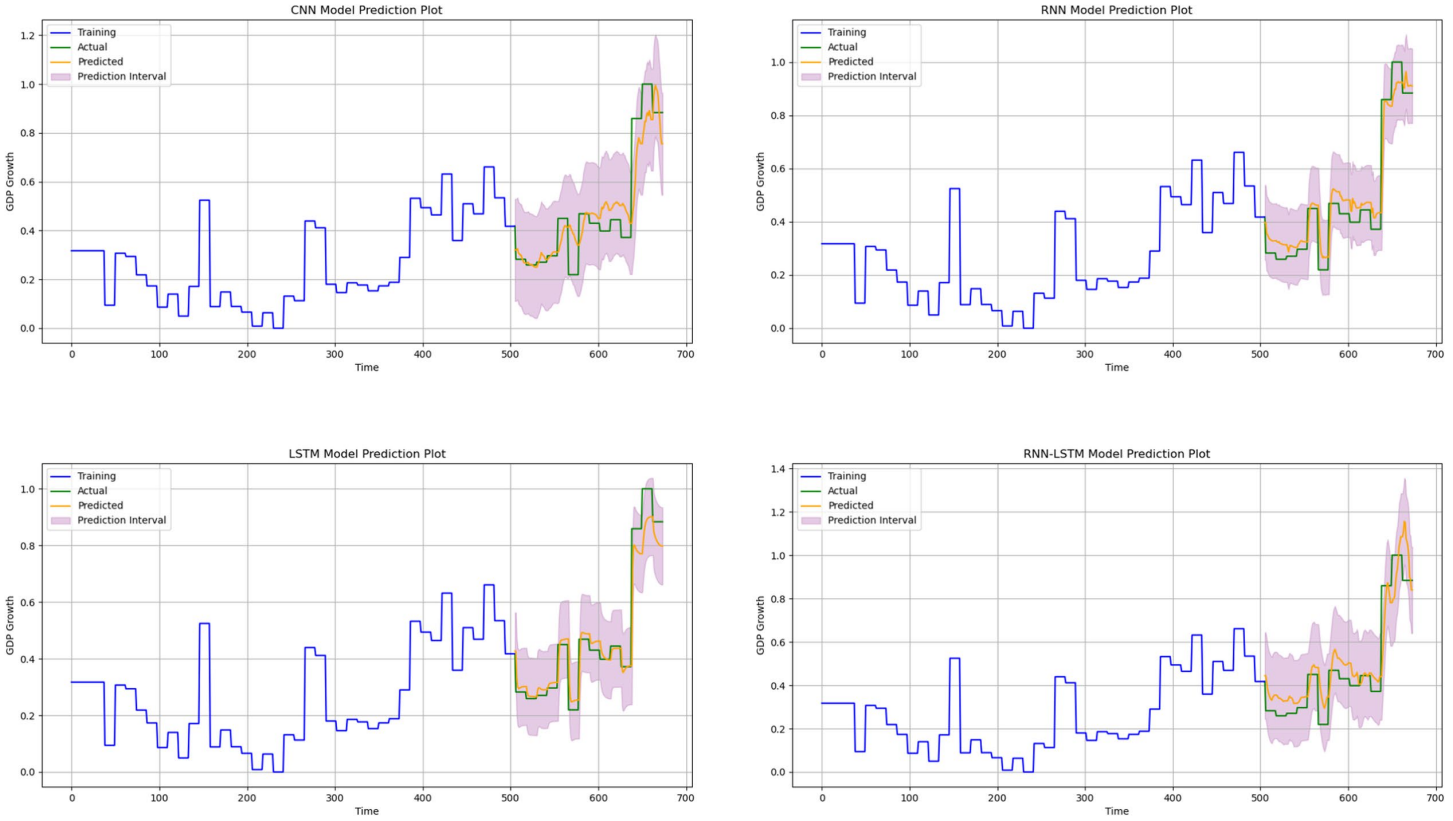
Comparison between the predicted values and actual values for our models. The upper-left sub-figure shows the CNN model and upper-right sub-figure shows the RNN Model. While the lower-left sub-figure shows the LSTM model and the lower-right sub-figure shows the hybrid RNN-LSTM model.

Furthermore, we used the prediction interval in evaluating the model’s prediction since predicting the time series problem involves uncertainties. The prediction interval provides valuable information about the uncertainties associated with the estimates. According to Figure 11, the RNN-LSTM hybrid model outperformed the other models. We conducted our analysis from three viewpoints: the trends, the differences between the actual values and predicted values and predicted interval. When using the hybrid RNN-LSTM model, the predicted values were consistently aligned with the actual values in both trend patterns. Specifically, when the actual GDP growth based on remittance inflows increased, the predicted values also increased, and when the actual values decreased, the predicted values followed the same pattern. It is worth noting that during significant turning points, both models were able to capture the changes in GDP growth based on remittance inflows trends effectively. However, predicted values from the RNN- LSTM hybrid model were much closer to the actual values. The predicted interval for the RNN-LSTM hybrid model were much closer too compared to the other models. Therefore, we can reasonably say that the RNN-LSTM model is a reliable tool for predicting GDP growth based on remittance inflows. The comparative results of the models, presented in Table 8 and Figure 11 support our assertion that overfitting is not a significant issue. These visual aids provide evidence of the model’s performance and validate our strategies to address potential overfitting concerns. They also demonstrate the model’s performance metrics and generalization ability, thus strengthening our conclusions.

Our outputs also suggest that our hybrid RNN-LSTM model is more computationally optimized in training compared to the other three models. Moreover, incorporating transfer learning and fine-tuning hyperparameters contributed to enhancing the model’s effectiveness. In summary, our hybrid RNN-LSTM base model had higher forecast accuracy and exceeded the performance of CNN, RNN, and LSTM base models in predicting GDP growth based on remittance inflow in The Gambia.

However, the proposed RNN-LSTM hybrid model has several limitations. Firstly, the model’s predictive performance heavily depends on the quality, relevance, and availability of the training data. In this study, the model was specifically trained and validated using datasets on economic indicators from The Gambia, including remittance inflows and associated macroeconomic factors. This geographic and economic context may limit the model’s applicability to other regions with different economic structures, requiring significant data adjustments and retraining to adapt it to new economic environments effectively. Furthermore, despite efforts to minimize errors through transfer learning and hyperparameter tuning, the model optimization process remains resource intensive. Employing search for hyperparameter selection was beneficial but computationally costly, as deep learning models like RNN and LSTM generally require substantial tuning to achieve optimal results. While transfer learning improved computational efficiency, additional hyperparameter tuning across numerous parameters, such as layers, neurons, dropout rates, and epochs, still demands considerable processing power and time. Another limitation lies in challenges inherent to RNNs and LSTMs, including the potential for vanishing gradients, which complicates the training of deep models over long sequences. While the dropout and early stopping techniques were implemented to address overfitting, these challenges persist, especially as the model’s architecture grows more complex.

Despite the encouraging performance metrics observed, limitations in the model’s generalizability and computational demands could hinder its scalability and adaptability in broader applications. Finally, the model may require recalibration when applied to regions with distinct remittance inflows, considering cultural and economic differences.

## 7 Conclusion and policy recommendations

GDP is essential for assessing the general condition of local, regional, and global economies. Remittances have emerged as one of the most significant topics today, particularly in The Gambia, warranting focused attention. This study proposes a novel model for estimating GDP growth based on remittance inflow in The Gambia, known as the Recurrent Neural Network-Long Short-Term Memory (RNN-LSTM). The LSTM component is employed to address the vanishing gradient problem encountered by convolutional RNNs. Besides that, standalone models such as CNN, RNN, and LSTM were chosen as comparison models to evaluate the predicting accuracy and stability of our RNN-LSTM model. Additionally, the study examined the effect of remittances on economic growth through a survey. The study employs time series data spanning from 1966 to 2022, sourced from the World Bank database and the Central Bank of The Gambia. The data was preprocessed to align yearly and monthly data by converting all yearly data to monthly, ensuring temporal consistency.

Based on the research outcomes, we can draw the following conclusions: The proposed RNN-LSTM hybrid model exhibits strong generalization capabilities, delivering stable predictions of GDP growth based on remittance inflow. Its performance significantly surpasses that of the CNN, RNN, and LSTM models. This study highlights the effectiveness of combining transfer learning with hybrid RNN-LSTM models for financial forecasting. In comparison to the other three models, the hybrid model demonstrated superior predictive accuracy and stability, achieving the highest R^2^ value of 91.13%. Our analysis from the survey indicates that remittance inflow shows a notable and positive correlation with economic growth, particularly in the short term. However, the study acknowledges the potential limitations posed by the quality of source data. While sourced from reliable institutions, economic datasets inherently include a random component and potential noise. Addressing this, the preprocessing steps and robust evaluation techniques employed in this study aims to ensure the reliability of the predictions.

While the study demonstrates the effectiveness of the RNN-LSTM hybrid model in predicting GDP growth with high accuracy, it is essential to recognize that model evaluation should not be limited to accuracy alone. Future research should also prioritize the explainability and robustness of machine learning models, as these factors play a critical role in ensuring safe and reliable decision-making in economic forecasting. This perspective aligns with the principles outlined in the” safe machine learning” framework proposed in recent studies (Giudici, 2024). By integrating explainability and robustness into model assessment, researchers can enhance the trustworthiness and practical applicability of machine learning in sensitive domains such as economic forecasting.

Therefore, we recommend that the government should encourage remittance recipients to channel funds into productive areas such as investments and human capital development. Additionally, introducing a “Diaspora Bond” could provide Gambians abroad with a secure, impactful investment option that supports national development. This bond could help finance key projects in infrastructure, healthcare, and education, directly contributing to economic growth. The government is also advised to create more job opportunities within The Gambia, which would help to retain skilled young individuals who might otherwise emigrate. Higher youth employment could reduce household reliance on remittances, allowing recipients to invest in businesses and save for their future.

In addition to remittance inflows, this study identifies population as another factor positively correlated with GDP growth, albeit to a lesser degree than remittances. The correlation analysis suggests that an increase in population size is associated with higher GDP per capita. This may be attributed to a larger labor force, increased domestic consumption, and broader economic activities that drive GDP growth. However, this relationship highlights the importance of leveraging population growth effectively through policies that enhance labor productivity, create job opportunities, and foster innovation. This ensures that population growth contributes sustainably to economic development.

In future research, we plan to test the model on datasets from diverse economic contexts to evaluate its adaptability across different regions. Furthermore, addressing the challenges of vanishing gradients and overfitting in RNN and LSTM networks will remain a priority. Techniques such as cross-validation, dropout regularization, early stopping, and gradient clipping will be explored to ensure stability and prevent overfitting. Additionally, future studies should focus on fine-tuning hyperparameters, exploring alternative optimization algorithms, and incorporating advanced AI techniques such as deep neural networks (DNNs) and support vector regression (SVR). Investigating the quality of source data through rigorous statistical analysis will be critical for validating the robustness of the models. Moreover, examining factors such as financial infrastructure, labor force participation, and government policies is essential for understanding the role of remittances in promoting sustainable growth. These factors significantly impact long-term GDP growth, making them crucial considerations for leveraging remittances effectively.

## Data Availability

Publicly available datasets were analyzed in this study. This data can be found at: https://databank.worldbank.org/source/2?country=IRN&l=en.
