# Supplementary material for: Transfer learning for predicting of gross domestic product growth based on remittance inflows using RNN-LSTM hybrid model: a case study of The Gambia
Source: Front Artif Intell. 2025 Feb 24;8:1510341. doi: 10.3389/frai.2025.1510341 (PMC11891165; doi:10.3389/frai.2025.1510341)
Supplement: Supplementary file 1 [file Table_1.doc]

**Appendix Table A.** Recent Methods for GDP Forecasting in Various Nations.

**Classification Author Method Nation Duration Outcomes**

Predictor Identification

and Model Design

Jallow H. et al. (2024)

RNN-LSTM hybrid Gambia 1966 to 2022 Achieved the highest

R^2^ score of 91.285%

[(Shams et al.,](#_bookmark66) [2024)](#_bookmark66) PC-LSTM-RNN

(Pearson Correlation LSTM RNN)

[Lai](#_bookmark49) [(2022)](#_bookmark49) Three-stage multi- factor feature selection and deep learning

India 1961 to 2021 Achieved the highest R^2^ value of 99.99%

China 2000 to 2022 Improved TCN prediction accuracy by over 10%

[Muchisha et al.](#_bookmark57) [(2021)](#_bookmark57)

Random Forest,

Ridge, LASSO, Elastic Net, SVM

Indonesia 2013 to 2019 Random Forest

outperformed other models

[Qureshi et al.](#_bookmark60) [(2020)](#_bookmark60) Extreme Gradient

Boosting (XGBoost)

[Cicceri et al.](#_bookmark30) [(2020)](#_bookmark30) Nonlinear

Autoregressive

with Exogenous Variables (NARX)

Canada 2004 to 2019 RMSE: 0.019763,

MAE: 0.016845,

MSE: 0.019763

Italy 1995 to 2019 MSE: 0.079,

Accuracy: 0.87

[Maccarrone et al.](#_bookmark54) [(2021)](#_bookmark54)

K-Nearest Neighbour (KNN), time series

United States 1976 to 2020 KNN captures self-

predictive ability better than time series models

[Hossain et al.](#_bookmark41) [(2021)](#_bookmark41) Random Forest

Regressor

Bangladesh 1980 to 2019 MSE: 0.004, MAE:

0.062, RMSE: 0.068

[Jahn](#_bookmark47) [(2020)](#_bookmark47) Artificial Neural Network regression

24

Industrialized Economies

1992 to 2017 Artificial neural

networks outperform conventional panel models

Sequential Data Forecasting

[Velidi](#_bookmark73) [(2022)](#_bookmark73) LSTM and RNN Indonesia 2018 to 2022 Achieved accuracy

of 80%-90%

[Lai](#_bookmark49) [(2022)](#_bookmark49) Particle Swarm Optimization (PSO) and Elman NN

China 1992 to 2020 MAPE: 0.0236,

RMSE: 0.0166

[Longo et al.](#_bookmark52) [(2022)](#_bookmark52) RNN, DFM-GAS United States Post-2008-09

crisis

RNN and DFM-GAS

improve forecasts

[Richardson et al.](#_bookmark62) [(2021)](#_bookmark62)

Various machine- learning algorithms

New Zealand Real-time

(ongoing)

Significantly improve nowcasting performance

[Abonazel and Abd-](#_bookmark19) [Elftah](#_bookmark19) [(2019)](#_bookmark19)

ARIMA Egypt 1965 to 2016 MSE: 0.0076

[Ortega-Bastida et al.](#_bookmark59) [(2020)](#_bookmark59)

AutoEncoder, NC Filtered, e-SVR

Spain 2012 to 2016 RMSE: 0.360, MAE:

0.25
